# A review of autobiographical memory studies on patients with schizophrenia spectrum disorders

**DOI:** 10.1186/s12888-019-2346-6

**Published:** 2019-11-14

**Authors:** Yujia Zhang, Sara K. Kuhn, Laura Jobson, Shamsul Haque

**Affiliations:** 1 Department of Psychology, Jeffrey Cheah School of Medicine and Health Sciences, Monash University Malaysia, Jalan Lagoon Selatan, 47500 Bandar Sunway, Selangor Darul Ehsan Malaysia; 2 Department of Psychology, University of North Dakota, Grand Forks, North Dakota USA; 3 Turner Institute of Brain and Mental Health and School of Psychological Sciences, Monash University, 18 Innovation Walk, Clayton Campus, Wellington Road, Melbourne, Melbourne, VIC 3800 Australia

**Keywords:** Schizophrenia, Autobiographical memory, Cuing methods, Self-memory system, CaR-FA-X model

## Abstract

**Background:**

Patients suffering from schizophrenia spectrum disorders demonstrate various cognitive deficiencies, the most pertinent one being impairment in autobiographical memory. This paper reviews quantitative research investigating deficits in the content, and characteristics, of autobiographical memories in individuals with schizophrenia. It also examines if the method used to activate autobiographical memories influenced the results and which theoretical accounts were proposed to explain the defective recall of autobiographical memories in patients with schizophrenia.

**Methods:**

PsycINFO, Web of Science, and PubMed databases were searched for articles published between January 1998 and December 2018. Fifty-seven studies met the inclusion criteria. All studies implemented the generative retrieval strategy by inducing memories through cue words or pictures, the life-stage method, or open-ended retrieval method. The Preferred Reporting Items for Systematic Reviews and Meta-Analyses (PRISMA) Statement guidelines were followed for this review.

**Results:**

Most studies reported that patients with schizophrenia retrieve less specific autobiographical memories when compared to a healthy control group, while only three studies indicated that both groups performed similarly on memory specificity. Patients with schizophrenia also exhibited earlier reminiscence bumps than those for healthy controls. The relationship between comorbid depression and autobiographical memory specificity appeared to be independent because patients’ memory specificity improved through intervention, but their level of depression remained unchanged. The U-shaped retrieval pattern for memory specificity was not consistent. Both the connection between the history of attempted suicide and autobiographical memory specificity, and the relationship between psychotic symptoms and autobiographical memory specificity, remain inconclusive. Patients’ memory specificity and coherence improved through cognitive training.

**Conclusions:**

The overgeneral recall of autobiographical memory by patients with schizophrenia could be attributed to working memory, the disturbing concept of self, and the cuing method implemented. The earlier reminiscence bump for patients with schizophrenia may be explained by the premature closure of the identity formation process due to the emergence of psychotic symptoms during early adulthood. Protocol developed for this review was registered in PROSPERO (registration no: CRD42017062643).

## Background

*Schizophrenia spectrum disorders* (hereinafter referred to as *schizophrenia*) are chronic mental disorders with a lifetime prevalence rate of about 1% of the population worldwide [1, 2]. The symptoms of these disorders typically emerge between the mid-teens to mid-30s, with the disease onset in males being slightly earlier than in females [1, 3]. Patients with schizophrenia demonstrate significant cognitive deficiency, which is considered to be the primary reason for impairments in social functioning [4, 5]. Cognitive deficits in the domains of autobiographical memory (AM), working memory, processing speed, and cognitive control are prevalent [6–9]. Impairments in social cognition and theory of mind (ToM) are also widely reported [10, 11]. The aim of this paper is to review research examining one core cognitive deficit in patients with schizophrenia: deficits in AM. Specifically, we aimed to review the research that investigated: (a) deficits in AM content and structure; (b) how insight, depression, and history of attempted suicide affected the memory of patients with schizophrenia; and (c) theoretical accounts that were attributed to the defective recall of AMs among these patients. Studies in this area implement different cuing techniques; thus, a secondary aim was to consider whether AM methodology influenced results.

We are interested in the AMs of patients with schizophrenia because deficits in this type of cognition significantly impair an individual’s self-identity, ability to learn from experiences, and social communication. The most recent systematic review on AM in patients with schizophrenia summarised findings concerning the components of AM among patients with schizophrenia, and other mental disorders, in the case where AM has been studied [12]. This comprehensive review included 78 full-text articles selected from PubMed and PsycINFO and published since the inception of those databases through July 2015. The results illustrated that AM components such as form, content, awareness at retrieval, and lifespan distribution were impaired in patients with schizophrenia compared to normal controls. The reviewers concluded that defective AM is a major cognitive impairment in people suffering from schizophrenia [13].

The current systematic review focuses on similar research questions, but also considers three additional and important aspects. First, it has re-examined various theoretical frameworks proposed to explain defective AMs in patients with schizophrenia, including the Self-memory System [14]. Second, as 14 new articles on the same topic have appeared in the literature since the publication of the last review, we included them in the current review to give an up-to-date overview of these findings. We also included an additional 15 articles published before the last review that were not previously considered by the reviewers. Third, since the previous review examined the intervention programmes used to improve patients’ AMs and found contradictory evidence, we aimed to offer an in-depth analysis of why some interventions worked and why some failed to produce any positive outcomes.

In this systematic review, features of AM in patients with schizophrenia were considered through the lens of the Self Memory System (SMS) [14] and the CaR-FA-X model [15]. According to SMS [14, 16], the retrieval of AM is guided by complex interactions among an individual’s working self, conceptual self, and the *autobiographical knowledge base*. The term “working self” in SMS refers to “a .complex set of active goals and associated self-images” [17]. The conceptual self, however, contains the socially constructed schema and categories that define the self, other people, and an individual’s typical interaction with the environment. It is learned through socialisation, schooling, and religious practices. The SMS model postulates that recall of AM requires activation by cues to access participants’ autobiographical knowledge base*.* Participants elaborate upon the given cue (often presented in the form of cue words) through mental imagery, which is then used to access the autobiographical knowledge base comprising lifetime period knowledge, general event knowledge, and event-specific episodic memory. Lifetime period knowledge refers to the general knowledge of significant others, common locations, actions, activities, goals, and plans that are characteristic of that period (e.g., *when I was at boarding school*) [14]. Lifetime period knowledge often represents thematic information in memory description, which can overlap with other thematic knowledge. General event knowledge is more specific. A general event represents a set of related events and contains different pieces of memories that can be grouped into the same theme (e.g., *I went to Penang with my friends*, *and we drove on the highway along the seaside and ate a lot of food that night*). The key feature of episodic memory is vividness, in which people form mental imagery of their past experiences. Vividness encompasses the sensory-perceptual details of an event, including sounds, smells, movements and emotions [14].

AM specificity depends on the *goals of working self*. The idea of “self-discrepancy,” coined by Higgins [18], is often used to interpret this process. A large self-discrepancy—which is the discrepancy found among the actual self, ideal self, and ought self—normally causes a negative feedback loop, while a small discrepancy results in a positive feedback loop [14]. These feedback loops have a direct effect on an individual’s current goals, intentions, or motives, which either inhibit or facilitate the effort to access the autobiographical knowledge base. If a positive feedback loop is formed, AM specificity is higher; in a negative feedback loop, specificity is reduced. A person’s ability to recall detailed past experiences indicates a high integration between goals and the working self [14]. Therefore, the less integrated the goals and working self, the less likely one is to access event specific knowledge; thus, more general (or lifetime period) knowledge is retrieved. According to Conway and Pleydell-Pearce [14], when there is an incongruity between the goals of working self and the autobiographical knowledge base, the SMS breaks down in terms of its normal functionality. If the SMS remains nonfunctioning, and the disagreement between these two components unresolved, symptoms of confabulation, disjunctions, or schizophrenic delusions may occur [14, 17].

Overgeneral AM is common among patients suffering from various mental disorders such as anxiety [15], depression [19], and post-traumatic stress disorder (PTSD) [20]. Deficiencies in social problem solving and feelings of increased hopelessness are associated with overgeneral AM [21]. Three hypotheses have been proposed to explain this phenomenon, collectively known as the CaR-FA-X model, and consisting of: the *capture and rumination* (CaR) hypothesis, *functional avoidance* (FA) hypothesis, and *impaired executive control* (X) hypothesis [15]. According to the capture and rumination hypothesis, people suffering from anxiety, PTSD, or depression, show a tendency to dwell upon events and thoughts which may monopolise working memory capacity, limiting the cognitive resources required to construct specific AMs [15]. Patients tend to ruminate about things that concern them. In a cued recall paradigm, they map the cues onto their current concerns rather than elaborate the cues adequately to initiate a search for a specific AM [22]. This mapping of cues and personal concerns results in the retrieval of abstract, self-related knowledge rather than specific AMs [23].

According to the functional avoidance hypothesis, patients retrieve overgeneral AMs to avoid negative affect linked to their traumatic life experiences [24]. The retrieval of detailed AMs, especially the traumatic ones, leads to distress among patients. Overgeneral AMs are negatively reinforced to protect them from excess suffering [25]. Lastly, the impaired executive control hypothesis suggests that construction of an AM requires the central executive of working memory to initiate and maintain the search within the autobiographical knowledge base [14]. Any kind of interference with this process—either by distracting attention or overloading working memory—would lead to early search termination and thus, overgeneral AMs [19].

A recent meta-analysis [13] conducted on 20 studies, which included 571 patients with schizophrenia and 503 comparison participants, revealed that patients had lower levels of AM specificity, provided less detailed memories, and reported less conscious recollection than healthy controls. A large-to-medium effect size was reported for those three AM parameters. The meta-analysis also examined different methods being used to induce AMs in the patient population and whether those methods influenced the findings. No evidence was found to suggest it did. Several theoretical explanations were provided for why patients with schizophrenia were less likely to construct specific AMs (i.e., AMs that are less specific in meaning, recall time, people involved, and location or setting), including difficulty retrieving events that occurred in less than 1 day. First, the authors of the meta-analysis—in line with the CaR-FA-X model—proposed that dysfunctional executive functions, due to prefrontal cortex dysfunctions at both structural and molecular levels, are responsible for the patients not being able to construct AMs with enough detail.

Secondly, patients’ impaired ability to encode AM details results in the retrieval of less specific AMs. Due to executive dysfunction, the patients face difficulty linking and organising details of the memory for future recall; a phenomenon known as *defective strategic encoding* [26]. These relational memory impairments described in schizophrenia [27, 28] point to defective functioning of the hippocampus [29]. A significant positive correlation was observed between left hippocampal volume and the number of AM details recalled [30], suggesting that alteration of the hippocampus may contribute to AM impairment observed in patients. However, it is quite difficult to examine the relationship between defective strategic encoding and AM specificity as personal life events normally take place several years before the AM test. In addition, the events occurring before onset of the disease—likely to be the beginning of the defective strategic encoding—also suffer from less detailed recollection. Thirdly, patients with schizophrenia often have traumatic histories and employ emotional regulation processes, such as cognitive avoidance, which may prevent them from recalling AMs with enough detail. Normally functioning people, [31–33] as well as people with high stress levels, [34] were also found to use these same emotional regulation processes.

The results summarised above from this published meta-analysis [13] are updated and expanded upon in the current review. For example, we have reexamined the types of memory activation methods being used in published studies. We aim to also address a range of other issues related to patients’ AM. First, although the meta-analysis examined if different cueing methods induced AMs with varying specificity, we have reassessed this aspect as several new studies have been published since the 2016 meta-analysis. Second, we were curious to know whether patients’ current level of insight, comorbid depression, and history of attempted suicide influenced their memory recollection. Third, we relooked at the distribution of patients’ AMs across the lifespan. Although this aspect was thoroughly reviewed by Ricarte and colleagues [12], we aim to discover if their results would be altered due to the recent publication of several new studies. Fourth, the theoretical accounts highlighted in the meta-analysis received limited support, so we have thoroughly examined the interpretations offered by various authors and summarised them. Finally, we examined literature reporting the effects of various intervention programmes on the improvement of patients’ memory specificity.

## Method

**Fig. 1:**
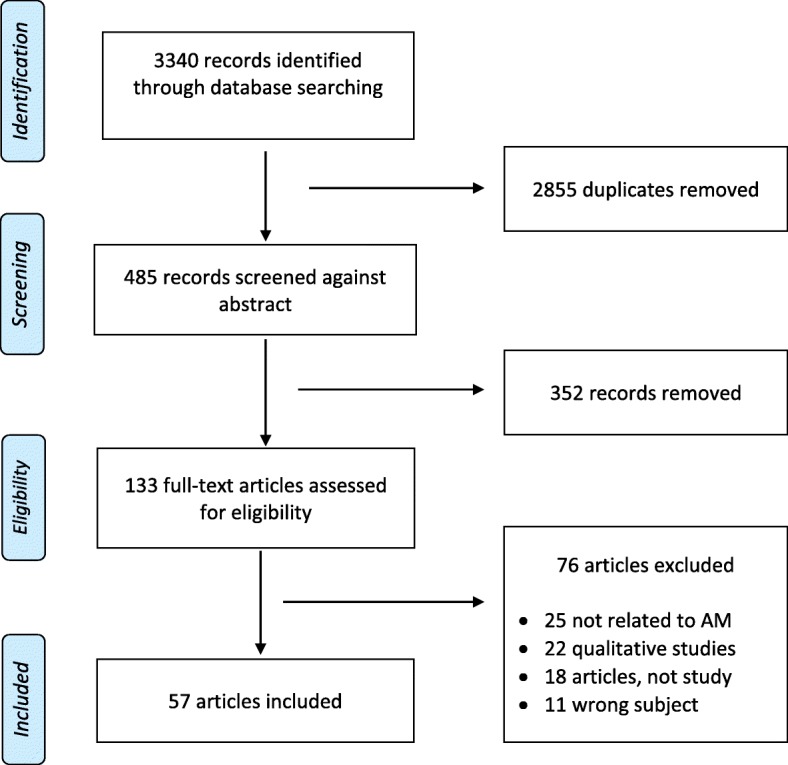
PRISMA-chart

Protocol developed for this review was registered in PROSPERO, an international prospective register for systematic reviews (registration no: CRD42017062643), and was executed according to the Preferred Reporting Items for Systematic Reviews and Meta-Analyses (PRISMA) Statement and flow diagram [35] (see Fig. 1).

The researchers searched PsycINFO, Web of Science, and PubMed databases for articles published between January 1998 and December 2018. Search terms were: “autobiographical memory”, “episodic memory*”*, “life story”, or “narrative”; and *“*schizophrenia*”*, “schizophrenia spectrum disorders”, “psychosis”, or “delusions”. Inclusion criteria were that studies must be quantitative, and: (a) published in an English language journal from 1998 to December 2018; (b) report on autobiographical memory (AM) data accumulated from patients with schizophrenia according to the Diagnostic and Statistical Manual of Mental Disorders (DSM-III, DSM-IV, and DSM V), or the International Classification of Diseases (ICD-9, or ICD-10) criteria; (c) compare AM performance among patients with schizophrenia, and also to healthy controls; and (d) report on at least one measure of features, content, or specificity of AM.

The first and last authors of this paper assessed article quality using 14 criteria developed by Kmet and colleagues [36], including: research objective, design, method, participant, measures, sample size, data analysis, results, and conclusion (see Appendix A). They assessed and scored each paper independently and assigned article scores from 0 to 2 reflecting the degree to which the specific criteria were met or reported on (i.e., *yes* = 2, *partial* = 1, *no* = 0). Items not applicable to a given criteria were marked *N/A* and excluded from the final calculation. Discrepancies between the two reviewers were resolved through discussion.

## Results

### Literature search

The initial database literature search for the current review produced 3340 titles, of which 759 from Ovid MEDLINE, 1240 from PsycINFO, 1338 from Web of science, and 3 from other sources. After screening the titles, 485 articles were retained as the duplicates were removed. A total of 352 articles were further removed from this list after screening the abstracts, keeping only 133 articles for full-text review. The screening of full-text articles excluded another 76 articles. The remaining 57 articles were finally included in the current systematic review, and their data were extracted (see Table 1).

**Table 1:** Extraction Tables for Selected Studies

Name of the Studies		Sample size	Assessment/Measure	Summary of findings	Interpretation	Quality check
1	Feinstein et al. (1998) [37] USA	Study 2: Patients, *n* = 19 Control, *n* = 10	• AMI*	1) When AMs, in terms of specificity, were distributed over the lifespan, patients exhibited a U-shaped curve, meaning more specific memories were recalled from childhood and recent years compared to early adulthood.	After initial failure in memory recall from early adulthood, some compensation may occur, allowing for better recall from later periods, as well as a “super-recent” effect. Also, childhood memory from the premorbid period was better encoded or more resistant to disruption.	0.818
				2) Controls, however, showed a relatively flat distribution.		
2	Kaney et al. (1999) [38] UK	Patients, *n* = 20 People with depression only, *n* = 20 Control, *n* = 20 Samples were matched in age and gender.	• AMT*	1) Patients generated less specific AM to positive cues than negative cues.	Reporting of overgeneral memory is a typical response while recalling a traumatic event. Patients avoiding giving specific details for their past events may be due to having problematic relationships in early life.	0.955
				2) Patients with delusional disorder generated less specific (or more categorical) AM than other groups.		
				3) Patients with depression generated more categorical AMs compared to other groups.		
				4) Patients with delusional disorder recalled AMs slower than controls.		
3	Elvevag et al. (2003) [39] Maryland	Patients, *n* = 21 Control, *n* = 21. Samples were matched in age only.	• Freely recollect episodes from participants’ lives.	1) Patients produced significantly fewer AMs than control participants.	Possibly “a general retrieval deficit” in the patient group. Patients’ performance deficit was due to encoding or acquisition problems during the most recent time period. The researchers believed that patients were left to their own devices; they did not use an efficient encoding strategy.	1.0
				2) Patients produced more AMs from the time preceding illness onset than after illness onset.		
4	Riutort et al. (2003) [40] France	Patients, *n* = 24 Controls, *n* = 24 Samples were matched in sex, age, and education.	• AME*	1) Patients showed an impairment of both components of AM (i.e., sematic and episodic memories) and a reduction of levels of specificity, compared to controls.	The drug treatment may have contributed to the patients’ retrieval patterns, like anti-Parkinson’s medication. Another reason could relate to a defect in encoding processes, provided that the illness was already present before the onset of symptoms. (i.e., neurodevelopmental hypothesis). Defective construction of personal identity may imply a developmental issue in the frontal lobe. SMS model: abnormality of personal identity, which was associated with reduced levels of specificity.	0.955
				2) Patients produced significantly less specific AMs than controls since the onset of symptoms.		
5	Corcoran et al. (2003) [41] UK	Patients, *n* = 59 Controls, *n* = 44 Samples were matched in sex and age.	• AMI*	1) Patients performed significantly worse than controls in all tasks.	Odd events in childhood, the reported reason for which could be potential traumatic childhood events.	0.909
				2) Patients tended to retrieve more odd AMs compared to controls.		
				3) The odd or negative recollections among patients tended to come from childhood.		
6	Harrison et al. (2004) [42] UK	Patients, *n* = 38	• AMT*	1) Patients recalled more categorical AMs compared to extended memories.	No theory or theoretical framework applied in explaining the results. However, the researchers mentioned that patients were recruited in different stages of recovery; therefore, there could be a sampling bias. These are limitations of the study, not an explanation for the findings.	1.0
				2) Patients’ depressive levels were significantly correlated with avoidance relating to psychosis.		
				3) There was a significant negative correlation between negative psychotic symptoms and retrieval of specific AM among patients.		
				4) Avoidance of traumatic memories relating to psychosis and specificity in AM were significant predictors for negative symptoms.		
7	Iqbal et al. (2004) [43] UK	Post-psychotic depressive patients, *n* = 13 Patients, *n* = 16 Samples were match in severity of illness, gender, education, and duration of symptoms or admission.	• AMT*	1) Patients with depressive symptoms recalled more general memory, especially with positive cue-words, than patient without depressive symptoms.	Authors believed that the severity of depressive symptoms was associated with positive or negative memories. Results were supported by the idea from Williams and Broadbent [15], that symptoms helped to avoid previous traumatic experiences (i.e., CARFAX Model- avoidance mechanism).	1.0
				2) No significance was observed between groups regarding specific memory retrieval.		
				3) Patients with depressive symptoms appeared to have better insight (i.e., awareness of illness) compared with patients without depression.		
8	Danion et al. (2005) [44] France	Patients, *n* = 21 Controls, *n* = 21 Samples were matched in age and education.	• ABME*	1) Patients rated significantly lower than controls in remembering AMs.	Researchers claimed that drug treatment could contribute to the current results. Moreover, according to the SMS, there would be a defect in executive processes (i.e., elaborative, strategic, and evaluative processes). The results reflected encoding or acquisition problems.	0.818
				2) Patients rated significantly more than controls to the events that they were unsure.		
				3) In terms of detail of AM, patients scored significantly lower compared to controls.		
9	Lysaker et al. (2005) [45] France	Outpatients, *n* = 52	• IPII* (with NCRS*)	1) Lack of awareness in patients was significantly related to narrative in terms of detail, temporal conceptual connection, and plausibility.	Authors did not provide any theory to support the results. However, they explained that positive symptoms will impact abstract/flexible thinking, hence it may have been associated with plausibility in the narrative. Poor plausibility in narrative reflected low function in socializing.	1.0
				2) Lack of awareness in patients was not		
				3) associated with positive or negative symptoms (i.e., neurocognition) and quality of life.		
				4) Lack of awareness was associated with poor performance in verbal memory.		
				5) Association between high levels of positive symptoms with poorer temporal conceptual connection, and plausibility in narratives was observed.		
				6) Plausibility in narrative was associated with quality of life (i.e., social function).		
10	Lysaker et al. (2005) [46]	Outpatients, *n* = 16 Control group: -Blind participants, *n* = 8 -MDD, *n* = 4	• Narrative interview (with STAND*)	1) The quality of narrative in patients was significantly poorer than controls in terms of self-worth (i.e., experiencing themselves as valuable to others) and agency (i.e., ability to sense that they can affect events in their own lives), indicating that patients consider their past to be of current social value, have a passive connection to others, and believe that their lives are controlled by outside forces.	Authors believed that negative symptoms (i.e., diminishing affect; volition) caused them to have little emotion; resulting in the telling of a thin story without much context.	1.0
				2) Negative symptoms and neurocognitive impairment were predictors of the poor quality of patient narratives.		
				3) No evidence that observed narrative disruptions were associated with levels of depression/anxiety or positive symptoms.		
				4) Awareness of illness was significantly associated with insight and judgement.		
11	Lysaker et al. (2005) [45]	Patients, *n* = 6 (average flexibility in abstract thinking); *n* = 10 (below average)	• IPII* (with NCRS*)	1) After a 5-month vocational rehabilitation, no significant result was observed in narrative coherence among patients.	Authors believed that patients with intact neurocognitive abilities may be able to develop more coherent stories after vocational rehabilitation, which provided patients with working experiences and helped them to gain a sense of identity and develop personal potential.	0.818
				2) However, the patient with average flexibility in abstract thinking seemed to improve in narrative coherence after 5-months vocational rehabilitation.		
12	McLeod et al. (2006) [47] UK	Patients, *n* = 20 Controls, *n* = 20 Samples were matched with age, education level, premorbid IQ, level of depression, and general memory ability.	• AMI* • AMT*	1) Patients showed a U-shaped temporal gradient, as they performed significantly worse in early adulthood than other periods of time (i.e., childhood and recent), in terms of personal facts.	Authors interpreted that patients’ AM retrieval process was aborted prematurely, possibly reflecting a general deficit of response inhibition. U-shaped pattern: patients’ disruption of encoding and consolidation process around the onset of illness (i.e., late adolescence and early adulthood). They speculated that patients’ encoding deficits may be presented from a very early age and then they remain stable even around the time of onset of illness. SMS: period of late adolescence and early adulthood is a time when AM knowledge is being organised to form a coherent personal identity. Goal of the *self* plays a major role in both the encoding and accessibility of AM in the normal population. Disturbing concept of self was considered a major factor in development of psychotic symptoms.	1.0
				2) Patients recalled recent personal facts better than childhood personal facts.		
				3) Controls did not show any difference in recalling events from three periods of life.		
				4) Patients recalled more recent events than events from childhood and early adulthood.		
				5) Patients produced significantly less specific and more categorical AMs, and more ‘uninterpretable’ responses than controls.		
				6) Among patients, the higher proportion of specific AMs was recalled on the AMT, and more AMs on the AMI.		
				7) Patients responded more quickly than the controls to both positive and negative cue words.		
13	Boeker et al. (2006) [48] Germany	Patients, *n* = 22 Controls, *n* = 22 Samples were matched in age, gender, education, and IQ.	• AMI* (life periods)	1) U-shaped retrieval style was observed.	Authors argued that lack of capacity in working memory and executive function may create an inability to maintain a coherent self. No further explanation provided regarding the AM in young adulthood.	1.0
				2) No significant difference between patients and controls in AM events and facts in youth and recent events.		
				3) Only significance occurred during young adulthood.		
				4) Correlation was not found among patients in executive functions and working memories, but was found for controls.		
14	Lysaker et al. (2006) [49] US	Patients, *n* = 64	• IPII* (with STAND*)	1) Higher level of narrative quality (i.e., illness awareness, agency, alienation) was associated with better social functioning and motivational hope among patients.	Patients’ positive symptoms may make them feel that they were not in control of their lives; hence a sense of self was interfered with.	1.0
				2) Positive symptoms were linked to lack of insight in illness, and an inability to affect events in their own lives.		
				3) Negative symptoms were also linked to lack of insight in illness and lower sense of self-worth.		
15	Cuervo-Lombard et al. (2007) [50] France	Patients, *n* = 27 Controls, *n* = 27 Samples were matched in age, education, and gender.	• To recall 20 autobiographical events in as much detail as possible.	1) Patients’ reminiscence bumps were earlier than for controls; patients’ bumps were for 16 to 25 years, whereas controls’ bumps were for 21 to 25 years.	Patients’ early bumps might be due to impaired personal identity, which is a fundamental disturbance in their illness. Defective retrieval processes may explain an overall reduction in performance among patients. Moreover, a defective sense of self may reduce the accessibility of highly self-relevant AMs. A defect in encoding or acquisition processes may account for the aggravation of AM abnormalities reported after the onset of the disease. Some AM abnormalities may merely be caused by the fact that patients had a poorer and more restricted life than normal subjects, and hence, encountered few memorable life events. SMS: Bump abnormalities in patients may be related to the formation of abnormal life goals.	0.955
				2) Patients rated significantly lower in AM specificity compared to controls.		
				3) Patients responded less in Remember; more in Know and Guess to AM details (i.e., what, where, and when).		
				4) Patients recalled fewer AMs related to births and deaths, but more related to work and education, compared to controls.		
16	Neumann et al. (2007) [51] Belgium	Patients, *n* = 20 Controls, *n* = 20 Samples were match in age, gender, and level of education.	• IAPS*	1) Patients performed worse than controls.	Patients’ response patterns could not be explained by drug treatment, as there was no correlation between IQ and memory performance. Remember responses reflected the level of confidence, not the recollected processes; hence, patients’ memories were weaker. Deficit in specific event knowledge in patients could explain their overgeneral retrieval pattern often associated with a simple feeling of familiarity.	1.0
				2) Patients recalled less AMs than controls.		
				3) Patients recalled less specific AMs, and more general memories compared to controls.		
				4) Patients recalled less negative AMs than controls.		
				5) Patients recalled more positive general memories, whereas controls recalled more negative specific AMs and more negative general memories.		
				6) No relationship was observed between psychosis symptoms and specificity of AM.		
17	Warren et al. (2007) [52] UK	Schizophrenic patients, *n* = 12 People with depression only, *n* = 12 Controls, *n* = 12	• AMI* • AMT*	1) Schizophrenic groups scored higher in anxiety and depression than controls. 2) Clinical groups generated more	Overgeneral memory retrieval is symptomatic of clinical conditions (i.e., depression; schizophrenia). It reflects a fundamental problem in memory processing. The researchers object to the view that overgenerality is caused by the avoidance of remembering personally relevant events, as patients retrieved fewer public memories than controls. Ruminative self-focus, associated with the pain of recalling specific events, can reduce working memory capacity; hence, block the search at the level of categorical descriptions. Also, researchers believed that past trauma might play a role in the development and maintenance of overgeneral retrieval of AM.	0.909
				3) categorical AMs than controls.		
				4) Depressed patients generated more specific AMs than patients with schizophrenia.		
				5) In terms of details of AMs, clinical groups did not differ.		
				6) Controls and depressive patients recalled AMs faster compared to patients with schizophrenia.		
18	D’Argembeau et al. (2008) [53] France	Patients, *n* = 16 Controls, *n* = 16 All subjects were matched in age, education, premorbid IQ, and levels of depressive symptoms.	• AMT*	1) Patients reported less specific and more categorical responses than controls.	Researchers claimed that deficit in patients’ AM was related to their ability to retrieve contextual information from memory and positive symptoms were associated with deficits in memory for contextual information. Patients’ deficits were in part related to disturbance of the sense of subjective time, which represents a key feature of episodic memory.	0.773
				2) Patients generated more specific responses for the past than for the future.		
				3) The proportion of specific responses generated by patients were negatively related to positive symptoms.		
				4) No significant correlation was found between negative symptoms and the proportion of specific responses.		
19	Blairy et al. (2008) [54] Belgium	Patients with AM intervention, *n* = 15 Control group patients, *n* = 12	• AMT*	1) Patients with AM intervention appeared to recall more specific AM before the intervention, and better performance compared with controls.	By applying treatment, patients’ capacity for retrieval was improved. No theory provided. However, authors argued that other than to improve patients’ AM, patients may need to acquire social skill to function better in daily life.	0.955
				2) No correlation observed between AM specificity (before and after intervention) and age, education, social functioning, illness duration, positive and negative symptoms, cognitive functioning, and level of depression or anxiety.		
				3) Positive correlation observed between AM specificity (before and after intervention) with executive function.		
				4) Follow up after 3 months: Patients with intervention recalled more specific AM than before treatment; however, no significant difference between after treatment and 3-month follow-up.		
20	Gruber & Kring (2008) [55] US	Study 1: Outpatients, *n* = 34 Inpatients, *n* = 8 Study 2: Outpatients, *n* = 24 Controls, *n* = 19 Samples were matched in gender, age, education, and marital status.	Study 1 • SDS* (without neutral life events) Study 2 • SDS* (with neutral life events)	1) Study 1: Patients’ narratives in negative stories were less clear, but had more detail, than positive events.	Authors suggested that patients were able to talk about their positive and negative life stories. However, they appeared detached from their emotional event stories, just like controls. No theories were mentioned.	1.0
				2) Patients’ narratives of positive events appeared more likely to involve other people, compared with negative events.		
				3) Study 2: No significant difference in terms of intensity of emotional life events between controls and patients.		
				4) Both groups used emotion words in the narrative context appropriately.		
				5) Less meaning clarity in positive and negative emotion narrative compared with neutral narratives among both groups, suggesting that both groups appeared to be detached.		
				6) Patients were as likely as controls to tell their pasts involving other people in a socially engaging manner		
				7) Patients’ emotion event narratives appeared less linear than controls in terms of temporal sequence.		
21	Lysaker et al. (2008) [56] US	Patients with: -Schizophrenia, *n* = 31 -Schizoaffective, *n* = 20 Samples were matched in age, education, and number of hospitalisations.	• IPII* (with STAND*)	1) Level of metacognitive (i.e., insight) was positively associated with education; not with age and history of hospitalisation.	Authors emphasised the importance of a biopsychosocial (i.e., medical, social, and psychological) approach for treating patients.	1.0
				2) Patients’ experiences of being		
				3) rejected were not linked to their quality of narrative.		
				4) Patients who performed better in metacognitive tests and had less internalised stigma tended to narrate better quality of their stories about their illness.		
22	Roe et al. (2008) [57] US	Patients, *n* = 65	• IPII* (with SUMD*)	1) Patients who denied symptoms and diagnoses in their story telling appeared to have lower levels of awareness of their illness (i.e., insight).	Authors explained about the current finding that patients held a range of beliefs and attitudes towards their illness, and the lack of insight might be a misinterpretation by researchers. Further studies may ameliorate this concern.	0.955
23	Lysaker et al. (2008) [56] US	Patients, *n* = 76	• IPII* (with NCRS*)	1) Patients with good insight into their illness in their narrative performed better regarding flexibility of abstract thoughts; better ToM (i.e., ability to understand other’s intentions and emotions).	Authors argued that patients with schizophrenia have a different level of awareness about their own psychological condition, which has an impact on their abstract thoughts and ability to understand other’s emotions and intentions. Moreover, their awareness of their condition contributed to their social functioning.	1.0
				2) Patients with superficial awareness (i.e., plausible life story with temporal coherency, but lacking in detail) were the same as patients with good insight in that they performed better in verbal memory and ToM than patients with poor insight into illness.		
				3) Patients with good insight or superficial awareness had more frequent social contact than those who had low insight.		
24	Raffard et al. (2009) [58] France	Patients *n* = 20 Controls, *n* = 20 Samples were matched in age, education, pre-morbid IQ, and levels of depression.	• SDMs*	1) No difference in recalling number of specific self-defining memories between patients and controls.	Patients present significant metacognitive deficits. The lack of meaning making plays a major role in the creation and maintenance of personal identity. Hence, fewer self-defining memories are centered on achievement themes. Patients’ reduced access to past experiences of success and increased access to episodes related to their illness contributed to maintaining a negative view of the self. Bump 10–20: concerns social identity formation. Bump: 20–30: concerns personal identity formation. Memories in the bump are self-defining as suggested by Conway and Holm.	1.0
				2) Patients produced significantly fewer integrated self-defining memories (i.e., meaning making) than controls.		
				3) Patients produced significantly fewer words in descriptions of self-defining memories than controls.		
				4) Patients produced fewer self-defining memories characterised by achievement content than controls.		
				5) Patients produced substantial amounts of self-defining memories characterised by hospitalisation/stigmatisation content.		
				6) The reminiscence bump peak for controls was 20–24 years; whereas for patients it was 15–19 years.		
25	Mehl et al. (2010) [59] Germany	Patients, *n* = 55 Controls, *n* = 45 Samples were matched in age, gender, and years of education.	• AMI*	1) Patients’ ability to infer emotions was positively correlated with ToM.	SMS: declarative cognitive tasks activate implicit AM, meaning that the ability to recall AM is more closely associated with the ability to infer intentions. Inferring emotion is associated with social performance, because emotion perception is an automatic process that requires procedural knowledge that is constantly refined while practicing it, in accordance with the model of skill acquisition.	1.0
				2) Patients were more impaired in the ToM ability to infer emotions.		
				3) Patients recalled less specific, less detailed, and fewer number of AMs compared to controls.		
				4) Patients’ deficits in AMs were associated with deficits in the ability to infer intentions.		
				5) Among patients, there was a significant association between AM (AMI total score) and social performance (total score).		
26	Taylor et al. (2010) [60] UK	Outpatients with history of suicide attempts, *n* = 40 Outpatients without history of suicide attempts, *n* = 20	• AMT*	1) Patients with past suicide attempts reported significantly higher levels of both depressive and anxious symptoms compared to non-attempters.	Researchers stated that patients’ retrieval style blocked access to potentially distressing specific memories, including those of traumatic and aversive experiences, as a way of self-protection. SMS: the retrieval style is tied up with one’s personal goals and self-identity. Memory systems may disrupt access to memories that are particularly aversive or ego-dystonic, so that patients avoid distressing memories.	1.0
				2) Traits of anxiety and depression were significantly related to AM specificity among patients.		
				3) Suicide attempters were more likely to recall specific AMs.		
				4) Negative cue-words were significantly associated with the highest proportion of distressing memories.		
27	Saavedra (2010) [61] Spain	Short length of stay inpatients, *n* = 9 Long length of stay inpatients, *n* = 9	• Narrative interview	1) Patients with a long length of stay appeared to have less delusion and lack of cohesion in their life stories compared to patients with short lengths of stay.	Patients with a long stay in the home setting, with serious paranoid schizophrenia, were able to narrate their lives.	0.909
				2) Long stay patients seemed to recall more stories related to relationships, activities, and illness.		
				3) Patients with long lengths of stay tended to use less words in describing negative life stories.		
				4) Longer stay patients used fewer words relating to the metaphysical (i.e., death; paranormal events), and had better inner organisation of narrative compared to patients with shorter lengths of stay.		
28	Pernot-Marino et al. (2010) [62] France	Patients, *n* = 8 Controls, *n* = 8	• Diary-recording	1) Number of Know responses associated with true and false memories was higher in patients compared to controls.	There was found to be a higher frequency of Knowing for false memories in patients possibly accompanied with a greater frequency of conscious recollection for false memories. Patients’ abilities to estimate the plausibility of events with respect to their current personal plans were impaired.	0.909
				2) The frequency of Remember responses was lower in patients than controls for true events		
				3) The frequency of Remember responses was higher in patients than controls for false events.		
29	Pettersen et al. (2010) [63] Norway	Patients with history of suicide attempts, *n* = 16 Patients without history of suicide attempts, *n* = 16	• AMT*	1) No difference between groups in current depression and hopelessness.	Cognitive functioning could be the reasons for the results, however, this study didn’t include any neuropsychological variables. Hopelessness often produces overgeneral AM, however, it was not observed in this study. Author argued that it could be due to limited statistical power.	0.955
				2) Patients with histories of suicide attempts reported significantly higher current suicide ideation compared to patients without histories of suicide attempts.		
				3) Patients with histories of suicide attempts produced significantly fewer specific AMs, and more general memories, compared to patients without histories of suicide attempts.		
				4) Patients with histories of suicide attempts showed a significant increase in number of specific AMs for negative and neutral cue words.		
30	Raffard et al. (2010) [64] France	Patients, *n* = 81 Controls, *n* = 50 Samples were matched in age, level of education, and premorbid IQ (i.e., National Adult Reading Test, Mackinnon & Williams, 1996).	• SDMs*	1) Patients did not differ from controls in the number of specific SDMs reported.	The earlier bump could be due to an impairment in immediate self-awareness and the disturbance of the temporal dimension of self that assures a stable and coherent sense of identity (the bump is a critical period for the formation and maintenance of a stable sense of identity). Life scripts is a schema of normative events that are culturally expected to occur at given times in the lifespan. However, patients might experience disruption to those expected normative events during illness onset, leading to isolation and social withdrawal.	1.0
				2) Patients demonstrated less meaning-making than controls.		
				3) Patients scored lower in achievement and relationship content than controls.		
				4) Patients recalled more life-threatening events than controls.		
				5) Patients recalled less coherency in context, chronology, and theme than controls.		
				6) Controls’ reminiscence bump peak was 20 to 24 years; patients was 15 to 19 years.		
31	Morise et al. (2011) [65] France	Patients, *n* = 18 Control, *n* = 17 Samples were matched in age and level of education.	• Chains of memories by using a personal memory as a cue for another memory.	1) The “chain” level explained 6.9% of the variance of emotional intensity score, and 15.7% of distinctiveness score in controls.	They believed that patients’ memory characteristics had lost their potential driving force in memory organisation, and that emotional experience associated with memories might play a compensatory role in respect to this organisation. AM impaired prefrontal activity and a dysfunctional connectivity between the amygdala and the prefrontal cortex.	0.818
				2) The “chain” level explained 15.1% of the variance of emotional intensity scores and 11.8% of distinctiveness score in patients.		
				3) The “chain” accounted for 26.9% of the variance of data in controls, and 36.2% in patients.		
32	Berna et al. (2011) [66] France	Patients, *n* = 24 Controls, *n* = 24 Samples were matched in age and education.	• SDMs*	1) 31% of self-defining memories in patients were related to a personal hospitalization and/or psychotic symptoms; 2.5% of the memories related to personal illness among controls.	The patients with more pronounced negative symptoms experienced severe impairments in giving meaning to their self-defining memories. Life altering events can have a profound impact on patients’ perception of themselves, especially in the context of a psychological disorder. The events become a landmark both for the self and in AM; consequently, influence personal goals.	0.909
				2) Patients’ meaning making was significantly lower compared to controls.		
				3) SDM was negatively correlated with level of negative symptoms among patients.		
33	Berna et al. (2011) [66] France	Patients, *n* = 24 Controls, *n* = 24 Samples were matched in age, education, premorbid IQ, current IQ, and self-esteem.	• SDMs*	1) 71% of memories from patients related to psychotic episodes; 29% referred to other past events having contributed to their illness.	The researchers believed that the reduced ability to give sense to illness-related memories does not seem to prevent these memories from positively integrating into the self.	0.909
				2) 15.6% of memories from controls related to personal illness and 84% to the illness of a close relative.		
				3) Patients produced lower meaning making than controls		
				4) Illness-related SDMs were more negative than other SDMs.		
				5) Patients displayed more traumatic memories than controls.		
				6) After splitting the patients into the ones with good insight and impaired insight, the results indicated no difference between them with respect to the proportion of events associated with redemption.		
34	Cuervo –Lombard et al. (2012) [67] France	Patients, *n* = 13 Controls, *n* = 14 Samples were matched in age, education, and verbal abilities (i.e., IQ).	• AMT*	1) No significant difference on the quantity of AMs and specificity scores among two groups.	The researchers stated that there was a decreased activation of the cognitive control network and aberrant activation of the dorsal striatum among patients.	0.864
				2) No significant differences in encoding age, remoteness, and valence between groups when retrieving memories.		
				3) Patients and controls activated a similar brain network during retrieval, including the cortical midline structures, left lateral prefrontal cortex, left angular gyrus, medial temporal lobes, occipital lobe, and cerebellum.		
				4) Patients displayed reduced activation in several of these regions compared to controls, including two cortical midline structures, the left lateral prefrontal cortex, left medial temporal lobe, occipital lobe, right cerebellum, and left lateral ventral tegmental area.		
35	Ricarte et al. (2012) [68] Spain	Patients, *n* = 24 (experimental group) Patients with Life Review therapy, *n* = 26 (active control group).	• ABME*	1) There was a significant negative correlation between pre-intervention BDI scores and semantic association (AM).	They explained greater impairment in AMs that were particularly marked for events that occurred after the onset of the disease. The improvement of mood was perhaps due to the intervention as one of the sessions focused on self-defining memories.	0.909
				2) All participants scored lower on conscious retrieval for the ABME in P3 and P4 compared to P1 and P2.		
				3) The experimental group displayed significantly increased AM specificity after the intervention.		
				4) Depression decreased after intervention in experimental group, but not in control group.		
36	Potheegadoo et al. (2012) [69] France	Patients, *n* = 25 Controls, *n* = 25 Samples were matched in age, gender, and level of education.	• ABME*	1) Patients’ self-esteem was significantly lower than for controls.	Patients’ perception of the subjective temporal distance (i.e. TD) of past events is distorted, causing them to reply more frequently on objective evidence than controls do when assessing the temporal distance of the past. This disturbance is particularly marked in the life period following the onset of schizophrenia. The amount of detail in patients’ AMs was not significantly correlated with the subjective TD of events. Authors argued that this lack of detail, temporal or non-temporal, represents one probable explanation for the distorted subjective time perception that has long been observed in schizophrenia.	0.955
				2) Patients provide significantly more objective explanations and responses without explanations.		
				3) Patients produced significantly fewer specific memories than controls.		
				4) Patients recalled significantly less temporal and non-temporal information than controls at P3 and P4.		
				5) Memory importance was higher at P3 than the other periods.		
37	Bennouna –Greene et al. (2012) [70] France	Patients, *n* = 25 Controls, *n* = 25 Samples were match in age, level of education, premorbid IQ, and current IQ.	• The Twenty “*I am* …” Statement Task	1) Patients appeared to have significantly lower AM specificity and Remember responses compared to controls.	Less specific and less consciously recollected AMs in patients were due to preserved temporal organisation (i.e., life-time period). Temporal organisation and characteristics of emotion was intact, but the thematic organisation and its distinctive features of memories were not. Patients depended not on distinctive characteristics of the event, but seemingly only on emotional intensity.	
				2) Patients had a lower proportion of active “I am” statements than controls.		
				3) Patients recalled significantly more negative AMs.		
				4) Patients were assessed lower in thematic link by experimenters.		
				5) “I am” statements: ✓ Explained 19.2% of the variance of the emotional intensity score; 28.3% of the distinctiveness scores in controls. But, 13.7%; 11.4% of emotional intensity and distinctiveness scores in patients, respectively.		
				✓ Patients only showed significant correlation between emotional intensity and thematic link.		
38	Herold et al. (2013) [30] Germany	Patients, *n* = 33 Controls, *n* = 21 Samples were matched in age, gender, and level of education.	• AMI* • E-AGI*	1) In patients, the volumes of the left and the left anterior hippocampus were negatively correlated with duration of illness.	Researchers claimed that long-term antipsychotic medication and their potential effects on brain structure must be considered as a potential confounding factor. Less frequently recalled memories may depend on hippocampal function.	0.864
				2) Patients reported significantly fewer episodic details than controls.		
				3) Episodic and semantic AM scores were not significantly correlated with age, duration of illness.		
				4) Semantic AM, but not episodic AM, was significantly correlated with education among patients.		
				5) Episodic and semantic AM were significantly correlated with left hippocampal volume in patients for both the left anterior and left posterior hippocampal.		
				6) There was a significant correlation between semantic AMs and both left hippocampal and left posterior hippocampal volume in controls.		
39	Potheegadoo et al. (2013) [71] France	Outpatients, *n* = 30 Controls, *n* = 30 Samples were matched in age, gender, IQ, and education.	• AMT*	1) Patients appeared to have high levels of depression, and lower self-esteem compared to controls.	Authors argued patients’ low specificity may be related to fewer responses related to Field perspective, suggesting that less specific AM could not allow the patients to re-experience their life stories.	0.955
				2) Specificity of AM in patients was significantly lower than controls.		
				3) AM specificity was higher when recollecting was associated with the Field perspective compared to the Observer perspective.		
				4) Among patients, no relationship was observed among levels of anxiety, depression, and self-esteem, and scores in the Field perspective. However, a significant relationship was observed between IQ and the Field perspective.		
40	Ricarte et al. (2014) [72] Spain	Patients, *n* = 31 Controls, *n* = 31 Samples were matched in age, gender, and years of education.	• AMT*	1) Patients scored significantly higher in depression and rumination than controls.	Car-FA-X model employed; 3 functions involved in the difficulties to access specific memories: 1) rumination, 2) functional avoidance, and 3) executive function. In this framework, executive function is essential during the effortful process of generative retrieval. AM impairment may not only relate to deficits occurring at the retrieval phase in patients. Dysfunctions in cognitive processes involved at the encoding phase or in the storage and organisation phase may also contribute to patients’ AM deficits.	0.955
				2) Patients retrieved fewer specific AMs than controls.		
				3) No significant correlation was observed between the number of specific memories and any other factors among patients.		
				4) Depression and rumination explained 24% of the variance of memory specificity in controls, but only 2.6% in patients.		
41	Moe & Docherty (2014) [73] US	Schizophrenic outpatients, *n* = 50 Bipolar patients, *n* = 17 Controls, *n* = 24	• Self-description	1) Quality of narrative was not associated with level of education.	Authors suggested that deficit in agency and relatedness to others were key factors among patients with schizophrenia.	1.0
				2) Significant results were observed between patients with schizophrenia and controls in the quality of narrative in terms of relatedness, level of self-definition, negative-positive self-regard, and self-critical nature, etc.		
				3) Moreover, significant results happened between patients with schizophrenia and bipolar patients in the quality of narrative (i.e., level of relatedness, level of self-definition, and negative-positive self-regard).		
42	Potheegadoo et al. (2014) [74] France	Outpatients, *n* = 25 Controls, *n* = 25 Samples were matched in age, education, IQ, and level of depression.	• AME*	1) Patients performed poorer than controls in all cognitive measures.	Authors concluded that specific cueing could help patients bring about more detail in emotion and cognition of their AM, however, it may not be enough to obtain richer information in sensory, temporal, and contextual information. It suggests that to elicit more sensory, temporal, and contextual details, requires a more complex encoding process.	1.0
				2) When the research provided less probing (i.e., nonspecific cueing phrases), lower specificity of AM was observed in patients and their AM appeared to have less detail and richness compared to controls.		
				3) With more probing (i.e., specific cueing) in sensory, temporal, contextual, emotional, and cognitive areas, patients’ AM was significantly lower in richness (i.e., sensory, temporal, and contextual), but not in details and specificity.		
43	Ricarte et al. (2014) [75] Spain	Patients, *n* = 16, with training Patients, *n* = 16, without training	• AMT*	1) Patients who received training showed a significant increase in AM specificity and detail.	Detailed and specific AMs can be learned through training among patients with schizophrenia. The hypothesis of rumination in the Car-FA-X model was employed to explain results; however, it could not fully illustrate the relationship between rumination and specificity.	0.955
				2) No significant change in depressive mood and ruminative thinking style before and after training for both groups.		
44	Herold et al. (2015) [76] Germany	Older patients, *n* = 25 Younger patients, *n* = 23 Controls, *n* = 21 All groups were matched in gender and education.	• AMI*	1) Older patients showed significant impairment in episodic and semantic AM compared to controls. 2) Older patients recalled fewer AM facts than controls.	The critical role of the hippocampus for recollection was emphasised by the researchers. Furthermore, they claimed that a progressive deteriorative course happened among patients.	0.864
				3) Episodic AM (but not semantic AM) was significantly correlated with education in the patient groups.		
				4) Hippocampal volume reduction in older patients, which is also detectable to a lesser extent in younger patients.		
				5) Hippocampal volume is significantly correlated with episodic and semantic AM in patients and controls.		
45	Buck & Penn (2015) [77] US	Inpatients, *n* = 66 Controls, *n* = 50 Samples were matched in age and gender.	• NET*	1) Controls used more words in their narratives compared to patients.	Authors concluded that assessing social cognition and language features in patient narratives may not be effective for identifying their impairment in social cognition.	1.0
				2) Patients used fewer words associated with negative symptoms; whereas patients using more words appeared to perform better regarding social functioning.		
				3) Patients used more pronouns in their sentences, especially personal pronouns (she, he, they, it, we, I, me, and them, etc.), and first-person singular pronouns (I; me).		
				4) No significant difference was observed for using positive words in a positive emotional context, and negative words in a negative emotional context.		
				5) Patients with a higher frequency for using first-person appeared to score lower in ToM.		
				6) Controls’ ToM was associated with negative emotion words and the use of first-person pronouns.		
46	Alle et al. (2015) [78] France	Outpatients, *n* = 27 Controls, *n* = 26 Samples were matched in age, education, and IQ.	• Life narratives	1) Patients scored significantly higher in anxiety and depression, and lower in self-esteem.	Authors believed that patients’ lack of coherence in stories was related to disorders of the self, lack of capacity in self-reflection, low levels of executive function, mental flexibility, or metacognitive issues.	1.0
				2) Patients narrated their life shorter than controls. However, there was no significant difference in vividness in their AM compared with controls.		
				3) In patients’ life stories, the emotional valence was less positive compared to controls.		
				4) Patients’ AM reasoning (i.e.,,meaning-making) and coherence of stories were significantly poorer.		
				5) No difference was observed in scores for culture life scripts.		
47	Buck et al. (2015) [79] US	Outpatients, *n* = 41	• IPII*	1) In patients’ narratives, their anticipatory pleasure was positively correlated with first-person plural pronouns (i.e., we, us, and our), and connection to the past in storytelling.	Socializing with others could make patients feel pleasure. Moreover, authors pointed out that the capacity to experience pleasure was related to one’s past; as seen in a meaningful way by the individual.	0.955
				2) Also, patients’ consummatory pleasure was positively correlated with first-person plural pronouns, and connection to the past in storytelling.		
				3) No significant relationship was observed between psychosis symptoms with the ability of experiencing pleasure.		
48	MacDougall et al. (2015) [80] Canada	Outpatients, *n* = 24	• To recollect 3 events in highly positive, highly negative, and neutral emotions.	1) Patients’ poorer performance in episodic memory for negative events predicted their lower insight (regarding social consequences) into their illness.	Authors suggested that AM performance predicted patient insight about their mental illness.	0.909
49	Alle et al. (2016) [81] France	Study 1: Outpatients, *n* = 20 Controls, *n* = 21 Samples were matched in age. Study 2: Outpatients, *n* = 30 Controls, *n* = 28 Samples were match in age, level of education, and IQ.	• Life narratives with free recall (Study 1) • Life narratives with structured protocol (Study 2): 7 important events.	1) Study 1: No difference between controls and patients in length of narratives.	Reduced global temporal coherence may be due to lower levels of executive functioning. Authors also emphasise the method of collecting life stories with a more structured protocol; global temporal coherence among controls and patients was enhanced.	0.955
				2) No difference between groups in terms of local indicators (i.e., date, age, life period, and distance from the present).		
				3) Study 2: Patients’ executive functioning was significantly lower than controls and their narratives were shorter.		
				4) However, patients’ capacities for identifying event order throughout the narratives (i.e., global temporal coherence) were poorer than controls, and there were more temporal distortion in patients’ narratives compared with controls in both studies.		
				5) With structured protocol, controls and patients appeared to score higher in global temporal coherence.		
				6) No difference was observed in temporal distortion and elaboration of ending in both studies.		
50	Holm et al. (2016) [82] Denmark	Outpatients, *n* = 25 Controls, *n* = 25 They were matched in gender, sex, and level of education.	• SDMs* • Life Story Chapters	1) Patients reported more negative life chapters; higher anxiety and depression than controls.	Neurocognitive functioning (i.e., including the self-reflection function) may be vital for constructing coherent life stories, and patients recruited in the current study were considered to be well functioning. Past traumatic events, loss of social interaction, awareness of their illness, and a high level of anxiety and depression could be reasons that patients rated their life stories negatively.	0.955
				2) Patients’ negative life chapters were not correlated with their depressive symptoms.		
				3) No significant difference in causal coherence, self-continuity and centrality to identity between patients and controls.		
				4) Patients with good performance in cognitive tests have higher causal coherence of life story chapters and SDMs.		
51	Alle et al. (2016) [83] France	Outpatients, *n* = 27 (no depressive symptoms, and IQ above 70) Controls, *n* = 27 Samples were matched in age, IQ, and level of education.	• Life narratives • CES*	1) No correction between self-continuity and executive function, self-esteem, and symptoms of illness	Metacognitive dysfunction could play a role in deficit in self-continuity (i.e., phenomenological continuity; narrative continuity). Past traumatic events could make patients rate their emotions as negative regarding their past experiences.	1.0
				2) Vividness of past events (i.e., phenomenological continuity) was lower in patients.		
				3) Patients appeared to have more negative emotions about the past than controls.		
				4) Patients appeared to have lower self-event connections compared with controls.		
52	Moe et al. (2016) [84] US	Patients, *n* = 47 Controls, *n* = 32 Samples were matched in age and gender.	• IPII* (with STAND*)	1) No differences between controls and patients in word-count for their narratives.	Authors suggested that idea density was more related to the narrative development to self-process instead of reflection in interactions with, or evaluations from, others. Patient illness might overtake the sense of identity and sense of self and turn them into self-stigma. Therefore, patients with greater idea density gained more insight; thereafter, more depression, anxiety, and lower motivation in life.	1.0
				2) Patients had lower idea density (i.e., account of information) in their narratives compared with controls.		
				3) Patients’ idea densities were not correlated with years of education or Global Assessment of Functioning.		
				4) Patients’ idea densities were negatively correlated with their positive symptoms; lack of flow in speech.		
				5) Their idea densities were also associated with levels of insight into their illness, and sense of control in life.		
				6) Patients were depressed and anxious tended to have higher level of difficulties with motivation.		
53	Holm et al. (2017) [85] Denmark	Patients, *n* = 25 Controls, *n* = 25 Samples were matched in age.	• SDMs*	1) Patients show earlier reminiscence bumps (i.e., 15–19) than controls (i.e., 25–29).	Authors explained that to obtain the diagnosis impaired the formation of SDMs. Moreover, patient’s early bump indicated that the period of obtaining the diagnosis happened during early adulthood. Furthermore, authors believed that the negative symptoms associated with patient’s fewer SDMs, and memories, may not be retained after the onset.	0.955
				2) 69% of patient memories were distributed over the years before diagnosis, whereas 27% of their SDMs were after diagnosis, and only 4% of memories were located within the year of diagnosis.		
				3) Patients with more negative symptoms appeared to recollect their memories less, especially after diagnosis.		
54	Willits et al. (2018) [86] US	Patients, *n* = 200 People with HIV as controls, *n* = 55 Samples were matched in age only.	• IPII* (applied Coh-Matrix)	1) Patients produced less speech in open-ended interviews, and more unique words compared to controls, suggesting patients’ tendency to jump from one topic to another.	Authors stated that results supported Bleuler and Jung’s ideas that people with schizophrenia have difficulties in connecting ideas, and they often tend to form a complicated narrative flow. Moreover, authors mentioned the disturbance in the sense of self, and metacognitive concerns related to their difficulties in constructing their life stories.	0.955
				2) Patients produced lower causal (e.g., because), logical (e.g., and), and contrastive (e.g., although) connections in their narratives, suggesting patients left others out to make sense of these essential links in their life stories.		
				3) Patients produced higher intentional content, and lower causal and intentional cohesion, indicating that patients often lack clarity when coming to both goal and non-goal activities with other people		
				4) Patients scored lower in deep cohesion compared to controls, indicating patients were less likely to provide links for people to understand their stories		
55	Alexiadou et al. (2018) [87] Greece	Patients, *n* = 40 Controls, *n* = 40 Samples were matched in age, gender, education, and IQ.	• QAM*	1) Controls performed better than patients in list learning, story recall, and verbal fluency.	Due to symptoms of disorder, patients had less of a social life, hence fewer memories to share. This might be related to the SMS. SMS: the working self takes the role of control in retrieving and encoding processes, and patients’ goals and intentions might be restricted, hence retrieving fewer specific memories. Functional avoidance: patients might have uncomfortable feelings when recalling their recent life events due to their life context (i.e., being hospitalised).	0.955
				2) Only recent life period patients performed worse than controls in recalling memories, after controlling variables for verbal memory and verbal fluency (by using hierarchical regression analysis).		
56	Nieto et al. (2018) [88] Spain	Inpatients, *n* = 24 Outpatients, *n* = 29 Controls, *n* = 69 Samples were matched in age, gender, and education.	• AME*	1) Patients experienced higher levels of depressive symptoms compared to controls, and their performance in working memory tasks was significantly worse than controls.	Metacognitive deficits could make patients have difficulty in interacting with others, hence they had fewer things to share about their lives. The high number of no reported memories from childhood in patients appeared to support the framework of the CARFAX model’s functional avoidance mechanism. Childhood trauma was considered a factor that contributed to later psychotic symptoms. However, authors acknowledged that since childhood trauma wasn’t measured, the finding of impaired specificity of memories during childhood should be reconsidered.	1.0
				2) Fewer memories reported by patients during childhood was significant compared to controls.		
				3) Those patients performing better in working memory tasks produce more memories during childhood, and more specific memories during early adulthood.		
				4) To recall AM during adolescence, patients with a higher number of sensations and emotions appeared to have lower scores for psychiatric symptoms.		
				5) Patients’ depressive symptoms were negatively correlated with number of specific AMs during the preceding year		
				6) Positive correlation observed between lack of childhood memories and psychiatric symptoms.		
				7) Patients recalled less specific AM, more general AM, and expressed fewer emotions compared to controls.		
57	Holm et al. (2018) [89]	Outpatients, *n* = 24 Controls, *n* = 24 Samples were matched in age, education, and level of depression.	• Narrative interview	1) No length differences between patients and controls in their stories.	Authors explained that patients in the current study seemed to have higher education and cognitive functions. The authors stated that cognitive function was important to generate temporal macrostructure or coherence. An unfulfilled need of agency and communion suggested a sense of not being control in one’s own life and a need for close relationships.	1.0
				2) No difference in number of chapters identified between groups.		
				3) No difference was observed in temporal macrostructure (i.e., agency and communion).		
				4) Both groups showed no differences in the extent of beginnings and endings of their stories. Both groups appeared to elaborate more in endings.		
				5) Patients had significantly less agency (i.e., need to be in control of one’s life) fulfillment.		
				6) Patients’ stories had significantly more unfulfilled communion (i.e., the need for intimate relationships, friendship, romance, sharing, and belongingness) themes.		

ABME*: Autobiographical Memory Enquiry

AME*: Autobiographical Memory Enquiry

AMI*: Autobiographical Memory Interview

AMT*: Autobiographical Memory Test

CES*: Centrality of Event Scale

E-AGI*: Erweitertes Autobiographisches Gedächtnisinterview

IAPS*: International Affective Picture System

IPII*: Indiana Psychiatric Illness Interview

MCQ*: Memory Characteristics Questionnaire

NCRS*: Narrative Coherence Rating Scale

NET*: Narrative of Emotions Task

QAM*: Questionnaire of Autobiographical Memory

SDMs*: Self-defining memories questionnaire

SDS*: Schedule for Deficit Syndrome

STAND*: Scale to Assess Narrative Development

We present results in four sections reflecting the four main themes extracted from the literature: (1) methods to activate AM; (2) features of AM retrieved by patients with schizophrenia; (3) enhancing memory specificity and coherence; and (4) theoretical accounts employed to interpret the results.

### Autobiographical memory activation methods

There were three activation/retrieval methods implemented in the reviewed studies: cue word/picture methods, life stages methods, and open-ended methods (see Table 2).

**Table 2:** Research Methods for Selected Studies

Generative retrieval type	Specific method or measure	Studies in chronological order
Cue word/picture method (17 studies)	Autobiographical Memory Test [90]	▪ Kaney, et al., 1999 [38]
		▪ Harrison & Fowler, 2004 [42]
		▪ Iqbal et al., 2004 [43]
		▪ Mcleod, Wood, & Brewin, 2006 [47]
		▪ Warren & Haslam, 2007 [52]
		▪ D’Argembeau et al., 2008 [53]
		▪ Blairy et al., 2008 [54]
		▪ Taylor et al., 2010 [60]
		▪ Pettersen et al., 2010 [63]
		▪ Cuervo-Lombard et al., 2012 [67]
		▪ Ricarte et al., 2012 [68]
		▪ Ricarte et al., 2014 [72]
	After giving a specific AM in response to a cue word, the participants were asked to give a title to the AM. This title was then used as a cue to evoke subsequent specific memories	▪ Morise et al., 2011 [65]
	International Affective Picture System [91].	▪ Neumann et al., 2007 [51]
	Narrative of Emotions Task (NET [92];), to provide simple (i.e., happy, sad), complex (i.e., suspicious, surprised), and self-conscious emotions (i.e., ashamed, guilty) to elicit their stories.	▪ Buck & Penn, 2015 [77]
	Participants were asked to provide three events in nature of highly positive, highly negative, and neutral stories. The scoring system from The Autobiographical Interview (AI [93];)	▪ MacDougall et al., 2015 [80]
	Schedule for Deficit Syndrome (i.e., SDS) [94]	▪ Gruber & Kring, 2008 [55]
Life stages method (14 studies)	Autobiographical Memory Interview (AMI [95];)	▪ Feinstein et al., 1998 [37]
		▪ Corcoran & Frith, 2003 [41]
		▪ Mcleod et al., 2006 [47]
		▪ Boeker et al., 2006 [48]
		▪ Mehl et al., 2009 [59]
	Autobiographical Memory Inquiry [96]	▪ Riutort, et al., 2003 [40]
		▪ Potheegadoo et al., 2014 [74]
	Autobiographical Memory Enquiry (i.e., ABME) [97]	▪ Danion et al., 2005 [44]
		▪ Ricarte et al., 2012 [68]
		▪ Potheegadoo et al., 2012 [69]
		▪ Potheegadoo et al., 2013 [71]
		▪ Nieto et al., 2019 [88]
	Erweitertes Autobiographisches Gedächtnisinterview (E-AGI Interview [98];) in 5 life time periods	▪ Herold et al., 2013 [30]
		▪ Herold et al., 2015 [76]
	Questionnaire of Autobiographical Memory (QAM), including 1) The Personal Semantic Memory scale (i.e., semantic AM), and 2) The Autobiographical Incidents scale (i.e., episodic AM), to access in 3 different life periods	▪ Alexiadou et al., 2018 [87]
Open-ended retrieval method (26 studies)	Free recall of 50 events from participants’ lives and association of dates to these events [99].	▪ Elvevåg et al., 2003 [39]
	Free recall of 20 important events from participants’ lives and attribution of ages and times for each event [100]	▪ Cuervo-Lombard et al., 2007 [50]
	Singer & Moffitt’s (1992) Self-defining memories (SDMs) questionnaire requiring participants to recall three important memories which help to define who they are.	▪ Raffard et al., 2009 [58]
		▪ Raffard et al., 2010 [64]
		▪ Berna et al., 2011a [66]
		▪ Berna et al., 2011b [66]
		▪ Holm et al., 2016 [82]
		▪ Holm et al., 2017 [85]
	Diary record [101]	▪ Pernot-Marino et al., 2010 [62]
	Kuhn & McPartland’s (1954) “Twenty-Statements” Test (TST) of *I am* statements	▪ Bennouna-Greene et al., 2012 [70]
	Life story chapters [102] requiring participants recall their life stories, and divide them into different chapters.	▪ Holm et al., 2016 [82]
	Life Narrative [103]: to recall 7 the most important past events, and narrate a story with those memories	▪ Alle et al., 2015 [78]
		▪ Alle, d’Argembeau, et al., 2016 [83]
		▪ Alle, Gandolphe, et al., 2016 [81]
	Indiana Psychiatric Illness Interview (i.e., IPII) [104]. IPII is to access one’s narrative with mental illness, including their life story, their understanding of illness, and lived experience of having such illness, and lastly how the illness control or being controlled.	▪ Lysaker, Davis et al., 2005 [45]
		▪ Lysaker, France et al., 2005 [45]
		▪ Lysaker, Buck et al., 2006 [49]
		▪ Lysaker, Buck et al., 2008 [56]
		▪ Roe et al., 2008 [57]
		▪ Lysaker et al., 2008 [56]
		▪ Buck et al., 2015 [79]
		▪ Moe et al., 2016 [84]
		▪ Willits et al., 2018 [86]
	Narrative interview: “tell a story of your life”, “describe yourself as fully as you can”	▪ Lysaker, et al., 2005 [46]
		▪ Saavedra, 2010 [61]
		▪ Moe & Docherty, 2014 [73]
		▪ Holm et al., 2018 [89]

#### Cue word/picture method

Seventeen studies used cue words or pictures to evoke autobiographical memories (AMs) from patients with schizophrenia (see Table 2). Cues included pictures or positive and negative emotion words (e.g., the words *happy* and *joyful* were used as positive cues; the words *insecure* and *lazy* as negative cues). The cue word/picture methods implemented in 12 studies were adapted from Williams and Broadbent [90]. The form (e.g., presented verbally or on cards) and number of cues in the reviewed studies varied. Several studies presented two cue sets (positive and negative) [38, 42, 43, 47, 54, 67, 72, 83], while others used three sets (positive, negative, and neutral) or defeat-related cues [52, 60, 63, 68, 80]. Sets of cue words employed varied. For example, McLeod and Wood [47] used *happy*, *proud*, *relieved*, *pleased*, *excited*, and *hopeful* as positive cue words, while Taylor and Gooding [60] used the words *tender*, *excited*, *friendly*, *peaceful*, and *pleasant*. Picture cues were also used [51], including 120 pictures (i.e., positive, negative, and neutral images) from the International Affective Picture System to stimulate participants’ emotions to evoke specific memories [91]. One study [65] used cue-within-the-cue words adapted from an earlier study [105]. After the patients retrieved a specific AM for each cue word, they were asked to title the memory; this title was used to evoke another memory in a subsequent trial [65]. One study [77] applied the Narrative of Emotion Task, asking participants to construct their experiences based on simple (i.e., happy; sad), complex (i.e., surprised; suspicious), and self-conscious (i.e., ashamed; guilty) emotion words. Another study [55] used the Schedule for Deficit Syndrome [94] to prompt AMs through the use of emotion words (e.g., happiness, enjoyment, sadness, upset, and irritation).

#### Life stages method

The life stages method was found in 15 studies. The Autobiographical Memory Interview (AMI) [95], employed in five studies (see Table 2), was the second most often used assessment. In AMI, participants are asked to recall three events in great detail from each life stage: childhood, early adulthood, and recent life. The Autobiographical Memory Enquiry (ABME) [97], the most frequently used measure, appears in six studies (see Table 2) to induce patients’ AM in four life stages: (a) childhood to age 9, (b) ages 10 to 19, (c) age 20 to 1 year before the test, and (d) the current year. The Autobiographical Memory Inquiry [96]—quite similar to ABME—appears in two studies (see Table 2), and also elicited the recollection of patients’ AMs in four life stages. The sole difference between these two measures lies in the representation of the second and third stages. The Autobiographical Memory Inquiry’s second stage is from age 11 to the age of symptom onset, and the third stage is from the age of onset to 1 year before the test [96]. Two studies (see Table 2) employed the German E-AGI (Erweitertes Autobiographisches Gedächtnisinterview) [98] to elicit AM recollection. It prompts memories for five life stages: preschool, primary school, secondary school, early adulthood (i.e., until the age of 35), and the most recent 5 years. One recent study [87] employed the Questionnaire of Autobiographical Memory (QAM), including two scales measuring semantic and episodic AM recalled from three different life periods.

#### Open-ended retrieval method

In 26 studies (see Table 2), open-ended retrieval methods encouraged participants to freely recall life events without providing more than necessary preconditioned information (e.g., at a certain period of time, or in a certain mood). The two most frequently used open-ended retrieval methods were the protocol from the Indiana Psychiatric Illness Interview (IPII) [104] and the method to recall self-defining memories (SDMs) [106]. While the former method was used in nine studies [45, 49, 56, 57, 79, 84, 86], the latter was used in six studies [58, 64, 66, 82, 85]. There were four sessions to complete the IPII. First, participants shared their life stories; next they described their psychological conditions, and thirdly, they shared their experience of having these conditions. Lastly, participants presented on how their conditions affected their lives. The SDMs used in six studies, required participants to recall three important memories to best define who they are and reflect their self-identities. Two additional studies [39, 50] asked participants to freely recall a number of past events and date them. One study [50] used a method [100] originally utilised by Holmes and Conway [100], in which participants freely recalled 20 important life events and provided an age and time for each.

Another open-ended retrieval method asked participants to keep a dairy. One study [62] encouraged participants to record four diary entries per day for 1 month, requiring participant motivation to complete this time consuming task for a successful study. Another study [70] employed the Twenty-Statements Test comprising “I am” statements to activate AMs and understand participants’ self-images and related AMs.

The Life Story Chapter [102] retrieval method was employed in one study [82] to elicit AM. It asks participants to recall their stories and divide them into chapters. Another three studies [78, 81, 83] used the Life Narrative method [103] which encourage participants to write down their seven most important events on cards, and then narrate them into a life story after arranging these cards in a temporal sequence.

### Features of autobiographical memory

#### Cue word/picture method

Most studies using a cue word or picture method revealed that patients with schizophrenia tended to report less specific AMs [38, 47, 52, 53, 72], fewer total memories [51], and more categorical memories [38, 42, 47, 52, 53]. However, one study [67] found no significant difference in specificity of AM between patients and controls. Patients and controls responded differently to positive and negative cues. One study [38] reported that patients provided more specific AMs for negative cue words and less specific memories for positive cue words. These results contradict another study [51] in which patients responded to positive picture cues with more specific memories. One study [55] revealed that patients reported less clear but lengthy descriptions of AMs in response to negative cues; yet their memories were more likely to involve others when responding to positive cues. Patient memory retrieval time varied when responding to cues. In two studies [38, 52], patients took a longer time to complete retrieval compared to controls, while in an opposing study [47], patients responded more quickly to cues.

Anxiety and depression levels were found to be higher among patients with schizophrenia than controls [52, 60], and these levels were positively correlated with avoidance relating to their illness [42]. Patients with depressive symptoms generated more general memories, especially towards positive cues; however, they seemed to have better insight regarding their psychological conditions than patients without depressive symptoms [43]. Additionally, patients’ impaired ability to produce more specific memories to negative cues predicted lower insight into their illnesses [80].

The relationship between patients’ comorbid depression and memory specificity is not conclusive. One study [60] indicates that patients’ depression levels highly correlate with memory specificity. However, two studies [54, 72] reported no such relationship. Risk of attempted suicide was high among patients with schizophrenia [1]. One study [60] revealed that patients with a history of attempted suicide reported higher levels of depressive symptoms and generated more specific AMs compared to patients without this history. Yet, another study, [63] reported that patients with a history of attempted suicide were more likely to produce less specific AMs compared to patients who have no such history.

Although patients with schizophrenia who take part in AM studies are generally stabilised by medication, their responses are occasionally “uninterpretable”. Thus, researchers find it difficult to make sense of how retrieval cues are related to the given memories for these responses [47]. Specifically, when negative cues are used, some patients’ memory descriptions are quite scattered [38, 60]. In one study [42], the recall of less specific AMs was associated with more negative psychotic symptoms in patients. Another study [53] reported that patients with greater positive psychotic symptoms were more likely to produce less specific AMs. However, one study [51] stated no such relationship.

Nevertheless, one study [54] notices that patients’ memory specificity is not associated with psychotic symptoms, social functioning, depression, or anxiety, but with their executive function. Patients with negative symptoms offered shorter descriptions of their stories; the more first-person pronouns they used, the poorer understanding they had about other’s emotions and intentions (i.e., ToM) [77].

Cue word/picture studies mostly support the notion that patients with schizophrenia recall less specific, and more categorical, AMs, and respond differently to positive and negative cues than controls. The link between the history of suicide attempts and AM specificity, and the relationship between psychotic symptoms and AM specificity, remain unclear.

#### Life stages method

Studies grouped into this category implemented the life stages method to evoke participants’ AMs. Findings were similar to those studies using cue word/picture methods. For instance, AMs among patients with schizophrenia appeared to be less specific [40, 41, 44, 59, 69, 71, 74, 88], and fewer in number [44, 59, 69], when compared with controls. This method, however, produced unique results. When patients’ AM specificity was distributed over the lifespan, “U-shaped” patterns appeared [37, 47, 48]. The patients’ AM retrieval was most impaired for early adulthood [37, 47, 68], and least impaired for childhood [37, 68] and recent years [47]. Research [47] suggests that early adulthood coincides with the onset of psychotic symptoms in many patients, resulting in less specific AMs. However, a recent study [87] has not confirmed these results, showing that patients’ memory performance from the recent years was poorer than for controls. Another study [88] found that the lack of memories from childhood was positively correlated with current psychotic symptoms.

When examining personal facts (i.e., semantic memories) in AMs, patients and controls demonstrated no difference [37]. However, patients performed better when retrieving recent personal facts than those from childhood [47]. The capacity for retrieving personal events (i.e., episodic memories) from all life periods was significantly impaired in patients compared to healthy controls [37]; however, opposing results from another study found that the deficit in recalling episodic memories only occurred in the recent life period [87] . One study [41] reported that patients tended to recall strange negative events from childhood, suggesting the possibility of having traumatic life experiences during childhood. Indeed, one review reported a causal relationship between childhood trauma, such as sexual and emotional abuse, and the patients’ current psychotic symptoms [107].

Researchers [68] revealed that mild depression correlated strongly with better memory recollection among patients with schizophrenia without retrieval training. They found that from 10 years old to the age of symptom onset (approximately 18–19 years), patients’ depression symptoms were lower while AM specificity was higher. This is consistent with the U-shaped retrieval pattern. After memory retrieval training [68, 75], patient memory retrieval improved, yet, there was no significant correlation between emotion and AM retrieval, indicating memory specificity levels were not related to changes in emotion (e.g., feelings of depression). Similar results were observed in another study [71] that found no relationship between depression and recollection.

Related to the ToM, one study [59] focused on how retrieval patterns illustrated that patients’ AM deficits were more strongly associated with a lack of ability to infer other’s intentions than to understand their emotions [108–110]. The researchers [59] indicated that patients with schizophrenia may have difficulty understanding other’s intentions or behaviors such as interpreting the meaning of conversations or body language. Another study [68] reported that patients’ capacities to retrieve past events were strong predictors of social performance.

One study [69] revealed that patients offered largely objective explanations of the importance of their memories, whereas controls provided mostly subjective explanations for their recalled memories. Older patients (i.e., mean age = 56) demonstrated significant impairment in AM (i.e., both episodic and semantic memories) compared to younger patients (i.e., mean age = 33) [76]. Two studies [47, 68] employed cue word and life stages methods to collect patient AMs. These studies revealed that patients recollected more specific events when given word cues and recollected a greater number of total events in the life stages method.

#### Open-ended retrieval method

Twenty-six studies [39, 45, 46, 49, 50, 56–58, 61, 62, 64, 66, 70, 73, 78, 79, 81–86, 89] used open-ended retrieval methods. Under this method, the studies were more likely to focus on the quality of AMs, such as meaning making, content, insight, self-worth, coherence, temporal conceptual context, plausibility, and so on. In addition, the results were similar to those that used cue word/picture and life stages methods. Patients with schizophrenia produced fewer memories than controls [39], and their memory specificity was lower [50, 70]. Additionally, patients produced more memories with high specificity preceding the age of psychotic symptom onset [39], supporting the U-shaped memory retrieval from the life stages recollection method [37, 47].

The patients recollected most of their memories from 22 years of age as opposed to 27 years of age for controls; and patients’ reminiscence bumps were also earlier [50]. Similarly, two studies [58, 85] reported patients’ reminiscence bumps to be temporally located from ages 15–19, and controls’ bumps from ages 20–24, or 25–29, respectively. Patients tended to recall more past events related to work and education than for births and deaths [50]. Controls were more likely to recall their past through intense emotional events [50], supporting other results [69] indicating that patients provide an objective explanation for important events while controls provide a subjective explanation.

One study [58] reported that patients with schizophrenia had a tendency to recall more memories relating to hospitalisation or stigmatisation rather than achievement, as compared with controls. A similar theme was found in one study [66] in which patients with schizophrenia tended to retrieve more traumatic memories than controls: 71% of these memories related to psychotic episodes while 29% related to other events that contributed to their disorders. Three studies [78, 82, 83] discovered that patients often rated their past as negative; however, recalling negative experiences was not associated with their level of depressive symptoms [82].

The content of memory produced by patients appeared to be less coherent regarding the context and theme. As far as the meaning making of events, studies revealed that patients produced less meaning making than controls [58, 64, 66, 78, 86]. Additionally, patients tended to use less temporal coherence [81] and less causal (e.g., “because”), logical (e.g., “and”), and contrastive (e.g., “although”) words in their storytelling [86]. But not all patient samples showed the same tendencies. Holm and colleagues [82] reported neither significant differences between controls and patients in terms of AM causal coherence and centrality to identity, nor the length of stories, their temporal coherence, number of life chapters, or themes produced [89].

Compared with healthy controls, patients’ AMs appeared to be lower in self-worth and agency [46, 73], meaning that they tended to connect with others passively, viewed the self as negative, and believed that their lives were not under their own control. These findings are supported by a recent study [89] stating that patients expressed their lack of fulfillment in agency and interpersonal relationships.

Patients’ insight into their own mental health conditions affected their AMs. Patients who denied the diagnoses or symptoms tended to have lower insight [57]. The patients with low insight produced less detailed AMs that lacked temporal conceptual connection and plausibility [45]. Vocational rehabilitation enhanced the AM coherence of patients with average insight, but did not for those with lower levels of insight [45]. Some patients with more insight seemed to have increased social functioning (i.e., ToM) [49, 56] and abstract thoughts [48], and were more motivated and hopeful [49] in life. However, others appeared to be more depressive and have lower motivation in life [84]. Patients’ poor levels of insight were associated with more psychotic symptoms [49] and with poorer quality AMs [107]. However, their AM quality was not associated with their past experiences of being rejected socially [56].

In terms of patients’ language, the more first-person plural pronouns (i.e., we, us, and our) used, the more anticipatory pleasure and greater connection with their past they experienced [79], indicating that socializing with other patients was pleasant. Patients who stayed longer in care homes produced stories that delineated relationships and home activities, and were more coherent and less delusional compared to the stories produced by patients whose stay in care homes was shorter [61]. In one study, patients were able to experience vividness of their past just like healthy controls [78], but an opposing result was observed in another study [83] in which the level of vividness was found to be lower in patients.

### Theoretical accounts

Various SMS schemes were employed in eight out of 57 reviewed articles to interpret why patients with schizophrenia often recall less specific AMs. According to these eight studies, this phenomenon of patient AM non-specificity might be due to the abnormality of personal identity [40], the disturbing concept of self [47], the goal of avoiding distressing memories [60], or unclear goals and intentions [47, 59, 87]. A defect in executive function was also suggested [44]. The unconventional reminiscence bump [50] and frequency of reporting inconsistent memories [62] in patients with schizophrenia was attributed to the abnormal formation of life goals. Additionally, vague descriptions were provided while applying the SMS framework; it was not made clear how the goals and identity of an individual could play a role in the memory retrieval process.

Only three studies employed the CaR-FA-X model to illuminate the possible reason for less specific AMs in patients with schizophrenia [43, 72, 75]. The first study [72] emphasised that the executive function is crucial during retrieval. The second study [63] stated that avoidance played a role in producing overgeneral AM among patients. The third study [53] employed the ruminative hypothesis in the CaR-FA-X model to explain the reduction in specificity in AM among patients, however, the result was not supportive to the theory as the patients’ ruminative thinking pattern remained unchanged although their AM specificity significantly improved through training.

Some studies highlighted the importance of working memory, executive function [48, 67, 81, 89], and metacognition [45, 56, 58, 64, 78, 80, 82–84, 86, 88] in explaining high quality AM (e.g., AM with specificity, internal coherence, length of narratives, and temporal conceptual context). A few studies mentioned that schizophrenia itself disturbed patients’ AM, especially the onset of the illness [68, 69] and its psychotic symptoms [45, 46, 49, 53, 66, 85]. Other studies stated that patients’ attitudes [57] or emotional states [63, 65, 70] had a significant impact on their memory retrieval.

Results from the remaining papers did not apply any theoretical framework but offered some general interpretations. Some authors discussed inefficient encoding strategies as the reason for patients recalling fewer AMs [39, 40, 50], while others emphasised the abundance of unpleasant life experiences [38, 41, 60] and general retrieval deficit [39, 47, 51]. Two studies mentioned that patients with schizophrenia follow an overgeneral memory retrieval pattern without much elaboration [52].

### Enhancing memory specificity and coherence

Several studies [45, 54, 68, 74, 75, 81] reported that patients’ memory specificity and coherence could be improved through both intervention and memory activation methods. Two studies from Ricarte and colleagues [53, 91], using different intervention and retrieval methods (i.e., life stage and cue-word), reported similar results that patient’s AM specificity and detail improved. However, the study [53], which employed Life Review therapy resulted in no change in patients’ moods or ruminative thinking patterns. The other study [91], which utilised event-specific training, resulted in differences in patients’ depression levels. These competing results are perhaps due to the intervention’s training objectives. Life Review therapy focused on positive past events, but did not make patients feel better afterwards. Event-specific training was more likely focusing on patients’ self-defining memories, which would be helpful in decreasing their depressive mood.

Blairy and colleagues [54] supported the results that AM specificity could be improved. They employed Cognitive Remediation therapy—exercising the recollection of memories through a written dairy. Although they were able to generate specific AMs, patients’ low moods remained. Holm and colleagues [89] discovered that patients reported greater unfulfilled communion, indicating that they wished to have close relationships or friendships. Future research could explore the correlation between patients’ moods and having close relationships.

Lysaker and colleagues [41] reported that narrative coherence of patients with an average level of insight was improved in a 5-month vocational rehabilitation programme. They believed that through working and interacting with others in the workplace, patients gained a sense of identity and recognised their personal potential, which then enhanced their narrative coherence. However, insight might be hard to obtain. One study stated that insight is culturally structured since patients’ insight levels are positively correlated with the insight of their family members [111].

Potheegadoo and his team [74] discovered that by adopting specific cues, patients with schizophrenia were able to recollect memories in detail with no difference in specificity from controls. The specific cues provided (e.g., “how did you feel when this event happened?”; “do you remember the thoughts you had at that very moment?”) might help patients to elaborate further while retrieving and constructing their memories. Patients might have difficulty expressing from their narrative exactly what researchers are looking for (i.e., a match with the scoring standard), as previous studies mention that patients’ ToM is weaker than controls [59, 68, 77]. More importantly, this study [74] highlighted an idea that the lack of specificity in AMs could be due to a lack of communication or elaboration skills, as the patients’ illness caused them to withdraw from social interactions.

Alle and colleagues [82] discovered similar results from adopting a more structured protocol to elicit patients’ AM (i.e., asking patients to write down the 7 most important events on 7 cards; narrate them into a full story after arranging them in temporal sequence). Either providing more specific cues, or a more structured protocol, would likely help patients with schizophrenia generate more specific memories. Future research could concern itself with the best method for accessing AM in patients with schizophrenia.

## Discussion

This systematic review has extracted four themes, which helped to answer our research questions. The first theme summarises all the memory activations methods commonly used in previous studies. The second theme highlights the features and content of patients’ AM together with the role of comorbid depression and history of suicidal attempt in memory specificity. The third theme synthesises the theoretical accounts of patients’ memory. The fourth theme summarises the ways by which one could enhance patients’ memory specificity and coherence. We discuss some additional findings on the relationships between psychotic symptoms and patients’ memory specificity.

### Memory activation methods

Research invariably shows that all studies used the generative retrieval strategy: giving cues to evoke autobiographical memories. The presentations of cues were in either words, pictures, life stages, or open-ended questions. Some results appear common across studies regardless of cuing method. For example, patients with schizophrenia retrieve less specific AMs compared to controls—only three studies [58, 64, 67] illustrated no difference in AM specificity, and one study [89] stated no difference in length, number of life chapters, or temporal macrostructure.

### Suicide attempts, comorbid depression, and AM specificity

Comorbid depression was common among patients with schizophrenia, with several studies reported patients having higher levels of depression compared to controls [52, 60, 71, 72, 78, 82, 88]. However, the relationship between patients’ level of depression and memory specificity was not clear. One study [60] indicated that patients’ depression levels were correlated with their memory specificity, while others showed no relationship between depression and memory specificity [54, 72], narrative disruption [46], negative life chapters [82], or memory recollection in the Field perspective [71]. It was also found that although patients’ AM specificity improved through intervention, their levels of depression remained unchanged [54, 75], indicating the independence of depression and memory specificity. Evidence shows that patients with depressive symptoms have better insight than those without depression [43]. Another study revealed that patients with higher levels of insight were more depressed; thus, were less motivated in life [84].

The patients with a history of attempted suicide generated more specific AMs compared to patients without such history [60]. However, another study showed that patients with a history of attempted suicide were more likely to produce less specific AMs compared to patients without this history [63]. These contradictory results could be attributed to the sample size and types of patients involved in the studies. While the first study recruited only outpatients (*N* = 60), the second study involved mostly inpatients (*N* = 23) and a smaller number of outpatients (*N* = 9). In a recent study, outpatients performed better in several neurocognitive tasks, such as processing speed, working memory, and attention, compared to inpatients [112]. Better neurocognitive function among outpatients could explain why they, despite having a history of attempted suicide, were able to produce more specific AMs than inpatients.

### Distribution of AMs across the patients’ lifespan

Studies [37, 39, 47] examining AM specificity illustrate patients having a U-shaped distribution, meaning they recollected more specific memories from childhood and recent years than early adulthood. However, the U-shaped retrieval pattern was absent in a recent study [87]. The lifespan distribution of patients’ typical AMs demonstrated an earlier reminiscence bump compared to healthy controls: 15–19 years for patients, and 20–24 years for functioning individuals [50, 58]. The participants’ average age at events was 22 years for patients and 27 years for controls. The early reminiscence bump for patients is probably due to the early onset of their illness; in many cases in their early adulthood, which may have resulted in the reduced recollection of memories from that period [113]. When functioning individuals were able to adequately encode events from early adulthood, the patients faced difficulty in doing so because of insomnia [114] and other neurodevelopmental abnormalities [115, 116]. As the patients were likely to show reduced awareness of the events taking place at a time when their disease symptoms started to surface, their memory recollection from that period dropped, resulting in the early closure of their reminiscence bump.

The identity account of the reminiscence bump [64, 117–119] could be employed to make sense of these findings. Normally, young people develop their self-identity during adolescence and early adulthood [120]. If an individual goes through a normal developmental process, there will be a privileged encoding of many of the events occurring during this period and later integrated with their lifelong narratives, thus enhanced accessibility to those memories later in life [121]. If an individual shows symptoms of schizophrenia during early adulthood, events occurring at that time would receive reduced attention and likely be disintegrated within their personal narrative. This is the reason for patients showing an inability to recall more memories from this period, resulting in an early closure of their reminiscence bump. The question that now arises what happens with the patients’ identity development process when they start experiencing schizophrenic symptoms for the first time. Does identity development pause during this period and recommence after a certain period when they start receiving medical care? Future research should focus on the seemingly uneven development of self-identity in patients with schizophrenia and how does that influence their memory recollection.

### Effects of psychotic symptoms on AM

A few studies have ascertained that psychotic symptoms relate to certain AM characteristics among patients with schizophrenia. Studies revealed that negative psychotic symptoms (i.e., social withdrawal; decreased or lost speech; emotional response) were related to patients’ AM specificity [42], self-defining memories [66], number of memories recalled after diagnosis [85], elaboration using fewer words [77], and negative symptoms, which predicted the poor quality of the narrative [46]. Since working memory was found to be a predictor of negative symptoms [122], a deficit in patients’ working memory might have affected their capacity to reminisce about their past.

Additionally, positive psychotic symptoms (i.e., hallucinations and delusions) were found to be related to AM specificity in one study [53]; however, the results were not corroborated in other studies [51, 54]. Furthermore, Alle and colleagues [83] discovered that the symptoms of illness were not related to self-continuity among outpatients (outpatients vs. inpatients appears to be an important variable in this research, but the type of patient samples are not clear in some studies). Also, positive symptoms appear to be associated with poorer temporal conceptual connection and plausibility in patient narratives [45], perhaps due to their low capacity for reality evaluation which is associated with positive symptoms [123]. A recent study [88] found that patients with fewer psychotic symptoms were likely to recall their memories from adolescence with more emotion and sensation.

### Theoretical accounts

The second research question was what kind of theoretical accounts were employed to explain overgeneral AM in patients with schizophrenia. Several interpretations were offered aligning with the SMS framework [14]. Primarily, patients’ overgeneral recall of AMs resembled General Event or Lifetime Period Knowledge as stated in the SMS. Issues such as abnormality in personal identity [40], disturbing self [47, 60], and unclear goals and intentions in patients with schizophrenia [47, 59] were held responsible for the less specific AMs. Impairments in executive function were also suggested to be responsible [44]. Although there was no explicit indication in any study currently reviewed, the goals of working self—according to the SMS of Conway and Pleydell-Pearce [20]—might play a key role in the lack of AM specificity.

The recall of less specific AMs indicates that patient goals of working self inhibit the construction of detailed memories by sampling information from the autobiographical knowledge base. This inhibition could be due to the discrepancy between the ideal self and actual self. There may be a large gap between knowledge of the past actual self and the perception of the ideal self. The larger the gap between selves, the more likely it is that a negative feedback loop is created [14], resulting in memory suppression or confabulation [17]. The discrepancy between selves could be rooted in the developmental history of patients with schizophrenia.

We have observed that participating patients in the reviewed studies accessed medical support at the time of data collection. Their psychotic symptom onset began earlier, approximately in their late teens or early adulthood [37, 39, 40, 65, 72, 124], a crucial period for adult identity development [125]. A struggle with psychotic symptoms at that time may have delayed the process of identity formation. Without proper support, training, or guidance, these individuals were not likely to function daily in work and relationships, causing a tendency to misinterpret others. Hence, it is inevitable that their developmental background, including a delay in adult identity formation, contributed to their goals of working self, which are affected by a significant discrepancy between selves.

The discrepancy between selves may results from the cuing method too, especially when emotional word cues were used to activate memories. The emotional words or pictures are more likely to induce discrepancy in participants’ current self and ideal self. Schoofs and colleagues reported that cues create self-discrepancy, causing the retrieval of less specific AMs by functioning student participants [126]. Similar findings were reported among elderly people [127] as well as patients with borderline personality disorder and depression [128]. There has been no study so far examining the relationship between self-discrepancy and overgeneral AM among patients with schizophrenia. This could be a focus of future studies.

Several studies suggested that people produce more general than specific memories because they want to minimise their self-discrepancy [126–129]. It is proposed that control of self-discrepancy is the nature of the goals of working self [14, 17]. The control of self-discrepancy is quite similar to the concept of a protective mechanism, as depicted in the functional avoidance hypothesis of the CaR-FA-X model. The protective mechanism functions to reduce discomfort and intense emotion [15]. There has been evidence demonstrating that unwanted memories might be directly suppressed, even causing later forgetting [130–133] among healthy people. As a few researchers report [134, 135], sharing non-specific memories is a type of self-defense mechanism among people with personality disorder. For example, in an extreme attempt to minimise the gap between selves, it is possible for patients to say: *I cannot remember*, or *I do not know,* as their goals of working self guide them to escape a situation and maintain self-coherence.

The CaR-FA-X model was used to explain less specific AMs in patients with schizophrenia [12]. In the reviewed articles, the executive function hypothesis was frequently employed for this purpose. This hypothesis suggests that to recall a specific and detailed AM, one has to initiate and conduct a conscious search within the autobiographical knowledge base which requires executive functions [14]. As the executive function is often impaired in patients with schizophrenia, they show reduced capability to construct specific AMs. The *capture and rumination* hypothesis was mentioned in only two studies reviewed. According to the *capture and rumination hypothesis*, patients with schizophrenia are often preoccupied with disorganised thoughts or intrusive images, causing them a high level of anxiety [136–138] or depression [138–140], which may take up a huge portion of their working memory capacity; resulting in reduced cognitive resources to form specific AMs [15]. The functional avoidance hypothesis did not receive any support from the studies we have reviewed.

Since the working self is crucial in the retrieval of AM and is incrementally shaped through the developmental process, examining patients’ upbringing would inform research by providing a better understanding of patients’ working selves. Although studies reviewed in this paper clearly show patients retrieving incoherent AMs, there is a dearth of evidence for the connection between cues dispensed and elicited memories. There is a potential gap between how patients and researchers make sense of patients’ reported memories; content labelled as uninterpretable may result from researchers’ judgments or perceptions.

Future research concerning AM in patients with schizophrenia could investigate their developmental stages, AM content, and sense making, particularly regarding their past. To attain these objectives, the life-story-interview method over the cue-word method seems better suited. Although the cuing method has some methodological advantages (e.g., consistency across participants), the life-story method can offer complete patient life narratives and highlight the most important influences, experiences, circumstances, issues, themes, and lessons of a lifetime [141, 142]. To ascertain how patients with schizophrenia make sense of their life and construct personal meaning and reality, the life story interview method is quite useful [143, 144]. This method is better able to show how patients organise, interpret, and create meaning from their life experiences, and how they maintain a sense of continuity.

### Memory enhancement in patients

Several studies have reported that patients’ memory specificity and coherence could be improved through structured interview protocols with specific cues, cognitive training, and rehabilitation [45, 54, 68, 74, 75, 81]. Interestingly, the memory coherence was only improved in patients who had at least a moderate level of insight [41]. When the interviewers asked very specific questions or presented with a specific cue, the patients were able to recall further detail of their life experiences. However, it is not made clear in the current studies if this memory enhancement was resulted from the cue itself or the process was moderated by a third factor, such as patients’ ToM. When more specific questions were asked, patients were able to realise what the interviewer’s expectation was; therefore, produced additional detail of their experiences. Future research should investigate the role of ToM regarding patients’ capability of recalling AMs. Various training and therapeutic interventions help to improve patients’ AM specificity, but the underlying cognitive processes deserve further examination. It is critical to understand if these interventions improve the patients’ self-perception and reduce their self-discrepancy, therefore positively affecting their memory recall.

### Strength/limitations

This systematic review has incorporated all the most recent empirical research on the AM of patients with schizophrenia. Unlike previous reviews, it has revealed that memory activation method itself plays a critical role in whether an individual would recall a specific or overgeneral AM. This review highlights how the SMS framework could explain overgeneral and incoherent AMs of patients with schizophrenia. This review has, however, a few limitations. It only included quantitative studies. Results from both qualitative and quantitative studies should be analysed for a more comprehensive understanding of the AM of people with schizophrenia. Another limitation is that some studies involved only outpatients, some only inpatients, and others utilised both. Even some studies did not clearly mention the type of patients they involved in their studies. Additionally, the patients participating in the reviewed studies were at different stages of recovery because they were all undergoing treatment. These factors may have affected the results reported.

### Directions for future research

Based on the reviewed research and extracted results, we offer some specific guidelines for future research. First, to see how patients with schizophrenia organise their past autobiographical knowledge, researchers could utilise the life chapters approach. This method has been used to investigate how patients suffering from major depressive disorder organise autobiographical knowledge within their past and potential future life chapters [145]. Future researchers could ask their patients with schizophrenia to list their past and future life chapters in a meaningful way (e.g., college education, job with HSBC) and sort a given list of positive and negative attributes according to those chapters. It would be interesting to see if the patients delineate their life chapters with more negative than positive attributes compared to healthy controls, and if there are any differences between attributes picked for their past and future life chapters. This kind of research could shed light on aspects of past knowledge that are associated with schizophrenic symptoms. Second, future studies could compare AMs of inpatients and those at different stages of recovery. Third, it would be interesting to see how AM relates to other cognitive processes (e.g., rumination, avoidance, problem solving) in this population and how AM problems contribute to and maintain symptoms.

Finally, we suggest cross-cultural research on AM in psychosis as previous research has shown that functioning individuals representing either collectivist or individualistic cultures report AMs differently [146, 147]. We also recommend future research on memory training approaches and mechanisms of change in emotions and symptoms. Some longitudinal studies could be conducted, as there are few such studies on patients with schizophrenia. It would also be interesting to compare content and appraisals of memories produced by patients with schizophrenia. We strongly recommend research investigating life scripts produced by this population and a comparison of their life scripts with the cultural life scripts of healthy controls.

## Conclusion

This review, following the PRISMA Statement guidelines, synthesised empirical findings published in 57 articles examining AMs in patients with schizophrenia. The features and content of AM, memory activation method, lifespan distribution of patients’ memories, and related aspects have been discussed. This review illustrates that most studies accumulated AMs through the generative retrieval process. Cuing methods such as words, pictures, life stages, and open-ended questions were used. Patients with schizophrenia were found to frequently recall more overgeneral memories. The patients who were still experiencing psychotic symptoms at the time of data collection were more likely to produce incoherent memories. While examining the lifespan distribution of specific memories, it was found that patients recalled more specific memories from childhood and recent years, but very few from early adulthood, forming a U-shaped distribution; however, this finding was not overwhelmingly consistent. Moreover, a typical reminiscence bump was found, but the patients’ bump started earlier than the bump exhibited by healthy controls. While patients exhibited the bump for 15–19 years of age, the controls exhibited it for 20–24 years of age.

The review indicates that patients’ AM specificity can be improved through retrieval training or by employing structured interview protocols with specific retrieval cues. Patients’ personal insights contributed to how they narrate their life stories, as their insight impacted on their mood. The relationship between history of suicidal attempt, level of depression and AM specificity was not conclusive; likely to be influenced by a third variable, such as patients’ stage of recovery, and patient type (i.e. inpatients or outpatients). Directions for future research to improve AM specificity in this clinical population were presented.

To explain overgeneral AM in schizophrenia, eight articles employed the SMS framework, three used the Car-FA-X model, and the rest did not use any theoretical account. The discrepancy between selves, dysfunctional working memory, unclear goals and intentions, and weaker ToM among patients, was held responsible for overgeneral AM. The reduction in recall of specific AMs from early adulthood was attributed to the lack of cognitive processing of the events due to the emergence of psychotic features during that period. Due to disease onset, events occurring during early adulthood were not processed to become integrated within the patients’ lifelong narrative, thus they were poorly recalled. Therefore, the earlier reminiscence bump for typical AMs in patients could be attributed to the early closure of the adult identity formation process.

## Data Availability

Data sharing is not applicable to this article as no datasets were generated or analysed during the current study.

## Abbreviations

ABME: Autobiographical Memory Enquiry
AM: Autobiographical Memory
AMI: Autobiographical Memory Interview
CaR-FA-X: *Capture and Rumination, Functional Avoidance, and* I*mpaired Executive Control*
DSM: Diagnostic and Statistical Manual of Mental Disorders
E-AGI: Erweitertes Autobiographisches Gedächtnisinterview
IAPS: International Affective Picture System
ICD: International Classification of Diseases
IPII: Indiana Psychiatric Illness Interview
PRISMA: Preferred Reporting Items for Systematic Reviews and Meta-Analyses
PTSD: Post-traumatic Stress Disorder
SDMs: Self-defining memories
SMS: Self-Memory System
ToM: Theory of Mind
